# Neural representations of Groups and Stereotypes using fMRI repetition suppression

**DOI:** 10.1038/s41598-019-39859-y

**Published:** 2019-02-28

**Authors:** Jeroen Delplanque, Elien Heleven, Frank Van Overwalle

**Affiliations:** Vrije Universiteit Brussel, Brussel, Belgium

**Keywords:** Social behaviour, Social neuroscience

## Abstract

Categorizing people in groups and associating them with stereotypical behavior is an integral part of human social understanding and interaction. This study investigates where knowledge on social groups and their stereotypes is represented in the brain. We presented participants with two sentences describing a group member (e.g. the police officer) performing a behavior believed to be stereotypical of the group (e.g. makes an arrest, i.e. authoritative), and asked them to rate the degree to which the behavior was typical of the group. Our critical manipulation was the repetition of this information across the two sentences: Either both the group and the stereotype implied by the behavior was repeated, only the group was repeated, only the stereotype implied by the behavior, or neither. Results showed robust suppression of hemodynamic activation from the first to second sentence in the medial prefrontal cortex in response to the repetition of the stereotype implied in the behavior, but only when groups were different. This finding suggests that the neural representation of stereotypes is located in this area, and this is in line with similar repetition suppression research showing trait representation in this area. A suppression effect for the repetition of groups was observed in the posterior cingulate cortex, regardless of whether stereotypes were repeated or not. This finding suggests that the neural representation of groups is located in this area. Because this location is unexpected, we discuss several suggestions for future research to confirm this finding.

## Introduction

Social categories and their stereotypes are influential aspects of our relationships with people. Our knowledge on social categories such as ethnicity, social status, religion, and gender often include over-generalized beliefs about them, called stereotypes^1^, and these beliefs help us to form impressions about these social groups. Since the seminal work by Allport^2^, research has consistently demonstrated the importance of social categories and stereotypes on impression formation and on our interactions with group members^3–9^. The use of stereotypes has clear advantages. It codifies our knowledge of groups^10^, it simplifies our social world^11^ and decreases our cognitive load during social interaction^12^. However, on the negative side, it can lead to stigmatization^13^ and discrimination^14^. Given the importance of groups in our everyday life, the purpose of the current study is to discover how knowledge about social categories and stereotypes is represented in our brain.

Several neuroscientific studies have shown that the mentalizing network is involved in the process of impression formation^15–18^. Key areas in this network consist of the medial prefrontal cortex (mPFC), temporo-parietal junction (TPJ), and posterior cingulate cortex (PCC, and nearby precuneus)^18–21^. These areas are responsible for inferring the mental states of others^19^, affective evaluations^22^, and trait judgments^23^. The mPFC in particular is associated with the processing of social agents, including social categories^24^, persons^25–28^ and the self^29^, as well as with their characteristics, including group stereotypes^10,30,31^, and personality traits^32,33^. Yet, the role of the mPFC in the representation of knowledge about groups and their stereotypes remains unclear.

The main question is whether information about groups and their stereotypes is held in persistent neural knowledge structures, which we refer to as a neural representation, or whether it is constructed on-line when encountering a group member. Holding this information in permanent neural representations might facilitate social reasoning and interaction, because this information is then rapidly available for use in social interactions to identify group members and to judge them. In favor of a persistent neural representation, prior research indicated a central role of the mPFC in making stereotypical associations about group members^10,30,31^. Researchers also demonstrated that analogously, we have persistent neural representations of persons^25–27^ and their traits^32,33^. Since groups are a congregation of individual persons, and stereotypes can be considered as a generalized attribution of a trait characteristic to a group, we can expect them each to be neurally represented in a similar manner as shown in this prior research. In other words, we would expect groups to be represented in a manner similar to persons, and stereotypes to be represented similar to traits. On the other hand, it is also possible that we do not possess such crystalized knowledge structures on groups and their stereotypes, unlike persons and personality traits. Arguing in favor of a transient construction process, some authors suggested that group impressions are created on-line, derived from contextually driven patterns of activity throughout the brain, rather than being represented in a specific region^34^. Such an on-line strategy might be more adequate and profitable to the extent that persons are members of multiple groups and social categories (e.g., male, Caucasian, Belgian, scientist, musician, etc.)^35^, so that group membership and judgments are largely determined by the interactional context rather than permanent representations that reflect our knowledge on groups and stereotypes. To test whether we hold persistent representations of groups, rather than make on-line constructions, an fMRI repetition suppression paradigm may be used.

The repetition suppression paradigm is based on the idea that if information processing leaves permanent memory traces, then information-processing is reduced when information is repeated, since it can make use of this memory residue^36,37^. This increased efficiency for repeated information is reflected in the brain by reduced activity in the area responsible for coding this information^36^, although it is still unclear which exact mechanism is responsible for this gain in efficiency^38^. Nevertheless, by this mechanism of repetition suppression, one can clearly isolate the neural representation of interest (e.g. personality trait), ignoring irrelevant neural activity caused by prerequisite preprocesses (e.g. behavior identification) or post-processes (e.g. emotional reactions) as revealed in traditional fMRI research^39^. This view is consistent with predictive coding^38,40,41^ and connectionist models of neural functioning^42^ that have also been applied to social cognition^43–46^ since suppression can be seen as a decrease in prediction error to the same stimulus in a memory code.

To identify neural representations, in earlier research on traits by Ma and colleagues^32^, two sentences were presented describing a protagonist performing a behavior. The two behaviors either implied the same traits, opposite traits, or the first behavior implied no trait (i.e. no repetition). Similar and opposite trait repetitions lead to suppression in the ventral part of the mPFC (vmPFC), but the condition without repetition did not^32^, indicating a neural representation for trait continuums in this area. A follow-up study documented that this trait suppression effect in the vmPFC was specific for personality traits, and not due to a general effect of valence^33^. The vmPFC does not only represent traits, however. The person performing this behavior is represented as well. Using a similar repetition procedure, researchers found suppression in the vmPFC when participants processed the same person engaging in trait-implying behavior, as opposed to different persons, for the same close other such as a friend or family member^27^, the same acquaintance such as a neighbor or colleague^26^, and the same unfamiliar person^25^. This suggests that persons are not only represented in this area for specific well-known individuals, but also for lesser or yet-unknown persons in a more generic format in order to facilitate their processing. Taken together, these studies clearly demonstrate the function of the vmPFC in representing relevant social information in an enduring knowledge structure for individual persons and their traits. Can these results be extended to the group level? Despite the prevalence of group thinking and social stereotypes in society, it is unclear whether there are also knowledge structures that firmly represent groups and their stereotypes in our brain.

To explore whether groups and their associated stereotypes are neurally represented, we used the same repetition paradigm as previous studies^32^. We presented sentences describing a protagonist as a member of a group (e.g., “The police agent”) who performed a behavior implying a stereotype (e.g., “made an arrest” implying authoritativeness). Two consecutive sentences referred to members belonging to the same or different groups, and the behaviors implied the same or different stereotypes. Since groups are a congregation of individual persons, and stereotypes can be considered as a general attribution of a trait to a group, we can expect suppression effects in regions that previously showed suppression for repeated traits or persons. We thus expected that repeated presentation of the same, compared to a different, group, stereotype, or both would reveal suppression in the mPFC.

## Method

### Participants

Participants consisted of 39 right-handed, neurologically healthy individuals (26 women) between the ages of 20 and 29 (M = 23.9). All participants were Dutch speaking graduate, or undergraduate students, with normal, or corrected to normal vision. Informed consents were acquired in accordance with the Medical Ethics Committee at the University Hospital of Ghent, where the study was conducted, and the Free University of Brussels. All experimental protocols were approved by these committees. In exchange for their collaboration, participants were compensated with 20 euros and travelling expenses.

### Stimulus Materials

All sentences in this experiment described a protagonist belonging to a specific social group performing a behavior. Each group had some sort of role in society, such as occupation (e.g. police agents, lawyers, and student), categories of age (e.g. the elderly, and teenagers), nationality, position in the family (e.g. housewives), etc. This ensured that a vast majority of the groups were necessarily outgroups. All groups and their stereotypes were created and selected using pilot tests. In a first pilot, participants (n = 20) were asked to identify groups together with their matching popular stereotypes. Based on these answers, a large set of sentences was constructed describing these groups and members’ stereotypical behaviors. In a second pilot, participants (n = 70) rated how stereotypical the behavior in the sentences was using a 7-point scale (with anchor points 1 = *not applicable at all* and 7 = *completely applicable*). All behaviors selected for the experiment implied stereotypes of positive and negative valence. Apart from the group name (e.g. “The police officer”), the length of the sentences varied from 5 to 12 words, with most sentences having 9 words. In total 400 sentences were produced and each was presented no more than once.

### Procedure

Participants were instructed to read the behavioral sentences and to infer the implied stereotype. After the participant was placed inside the MRI scanner the instructions were again displayed on the screen.

Each trial started with the words “Attribute of Group” in the middle of the screen for 2 s to remind the participant of the task. After this, two sentences (a prime and a target sentence) appeared consecutively on the screen for 5500 ms. Each sentence was preceded by a fixation cross with a variable duration jittered between 0 and 2000 ms randomly drawn from a uniform distribution. To keep participants focused on the task they were asked to rate how applicable the implied stereotype was in the last sentence, using one of four response buttons: 1 = *never* 2 = *sometimes*, 3 = *often*, and 4 = *always*. There were 20 trials in each condition. We presented one of five random blocks of the material to each participant, counterbalanced between conditions and participants. All trials were presented in a random order across conditions.

We created five conditions (see Table 1). In the Repeated Group/Repeated Stereotype condition, the protagonist in the two consecutive sentences was a member of the same group and the behavior implied the same stereotype. Stereotypes were considered the same if they implied the same generalized belief about the group. For instance, if two sentences both implied an authoritative police agent, the stereotype was considered to be repeated. On the other hand if one sentence described an authoritative police agent, and the other described a lazy police agent, the stereotype was not considered repeated. In the Repeated Group/Different Stereotype condition, the protagonist was a member of the same group but the behavior in the prime sentence implied a different Stereotype. The Different Group/Repeated Stereotype condition and Different Group/Different Stereotype conditions both involved different groups but had either similar or dissimilar stereotypes respectively. Finally, a Singleton condition was added in which only a target sentence was presented, in order to encourage the participants to also attend the first sentence.

**Table 1:** Overview of the conditions and sample sentences.

Condition		Prime sentence	Target sentence
Group	Stereotype		
Repeated	Repeated	A police officer gives a ticket.	A police officer makes an arrest.
Repeated	Different	A police officer helps the old lady cross the street	A police officer makes an arrest.
Different	Repeated	A train conductor gives a ticket.	A police officer makes an arrest.
Different	Different	A train conductor helps the old lady onto the train	A police officer makes an arrest.
Singleton		—	A police officer makes an arrest.

### Imaging Procedure

Images for the first 28 participants were collected with a Siemens Magnetom Trio scanner system (Siemens Medical Systems, Erlangen, Germany) using a 32-channel radiofrequency head coil. Images for the last 11 participants were collected with an upgraded Siemens Magnetom Prisma fit scanner system (Siemens Medical Systems, Erlangen, Germany) using a 64-channel radiofrequency head coil. Stimuli were projected onto a screen at the end of the magnet bore that participants viewed by the way of a mirror mounted on the head coil. Stimulus presentation was controlled by E-prime 2.0 (www.pstnet.com/eprime; Psychology Software Tools) running under Windows XP. Participants were placed head first and supine in the scanner bore. Participants were instructed not to move their heads to avoid motion artefacts. Foam cushions were placed within the head coil to minimize head movements. For the first 28 participants (in the Siemens Magnetom Trio scanner), first, high-resolution anatomical images were acquired using a T1-weighted 3D MPRAGE sequence [TR = 2530 ms, TE = 2.58 ms, TI = 1100 ms, acquisition matrix = 256 × 256 × 176, sagittal FOV = 220 mm³, flip angle = 7°, voxel size = 0.9 × 0.86 × 0.86 mm³ (resized to 1 × 1 × 1 mm³)]. Second, a fieldmap was calculated to correct for inhomogeneities in the magnetic field (Cusack & Papadakis, 2002). Next, whole-brain functional images were collected in a single run using a T2*-weighted gradient echo sequence, sensitive to BOLD contrast (TR = 2000 ms, TE = 35 ms, image matrix = 64 × 64, FOV = 224 mm, flip angle = 80°, slice thickness = 3.0 mm, distance factor = 17%, voxel size = 3.5 × 3.5 × 4.0 mm³, 30 axial slices). For the last 11 participants (in the upgraded Siemens Magnetom Prisma fit scanner), first, a high-resolution anatomical images were acquired using a T1-weighted 3D MPRAGE sequence [TR = 2250 ms, TE = 4.18 ms, TI = 900 ms, acquisition matrix = 256 × 256 × 176, sagittal FOV = 256 mm, flip angle = 9°, voxel size = 1 × 1 × 1 mm]. Second, a fieldmap was calculated to correct for inhomogeneities in the magnetic field (Cusack & Papadakis, 2002). Next, whole brain functional images were collected in a single run using a T2*-weighted gradient echo sequence, sensitive to BOLD contrast (TR = 2000 ms, TE = 29 ms, image matrix = 64 × 64, FOV = 224 mm, flip angle = 90°, slice thickness = 4.0 mm, distance factor = 10%, voxel size = 3.5 × 3.5 × 4.0 mm, 35 axial slices, acceleration factor GRAPPA = 2).

### Image Processing

The fMRI data were preprocessed using SPM12 (Wellcome Department of Cognitive Neurology, London, UK). Data were preprocessed to remove sources of noise and artefacts. Functional data were corrected for differences in acquisition time between slices for each whole-brain volume, realigned to correct for head movement, and co-registered with each participant’s anatomical data. The functional data were then transformed into a standard anatomical space (2 mm isotropic voxels) based on the ICBM152 brain template (Montreal Neurological Institute). Normalized data were then spatially smoothed (6 mm full-width at half-maximum, FWHM) using a Gaussian Kernel. Finally, the preprocessed data were examined using the Artifact Detection software package (ART, http://web.mit.edu/swg/art/art.pdf; http://www.nitrc.org/projects/artifact_detect), for excessive motion artefacts and for correlations between motion and experimental design, and between global mean signal and experimental design. Outliers were identified in the temporal differences series by assessing between-scan differences (Z-threshold: 3.0 mm, scan to scan movement threshold: 0.5 mm; rotation threshold: 0.02 radians). These outliers were omitted in the analyses by including a single regressor for each outlier. No correlations between motion and experimental design or global signal and experimental design were identified. Six directions of motion parameters from the realignment step as well as outlier time points (defined by ART) were included as nuisance regressors. We used a default high-pass filter of 128 s and serial correlations were accounted for by the default auto-regressive AR(1) model.

### Statistical Analysis

#### Behavioral Analysis

Two models were tested to map the effects on stereotype-rating and on reaction time (RT), respectively. In both cases a linear mixed effects model was used, which included a random slope for the participants. In the model with stereotype-rating as the dependent variable we tested the effects of RT and the five conditions. Conversely, in the model with RT as the dependent variable we tested the effects of stereotype-rating and the five conditions. Both models are reported using a type III Wald chi square test.

#### fMRI Analysis

Analysis of the fMRI data at the first (single participant) level were conducted using the general linear model of SPM12. The event-related design was modelled with two regressors for each condition (one for each sentence; there was only one regressor for the singleton condition), time locked at the presentation of the prime and target sentences and convolved with a canonical hemodynamic response function with event duration set to 0 for all conditions. Six motion parameters from the realignment as well as outlier time points (identified by ART) were included as nuisance regressors. The response of the participant was not modelled separately.

For the group (second level) analyses, we conducted a full factorial whole-brain analysis of variance (ANOVA) with conditions as within-participants factor, and coil as an additional between-participant factor, using a voxelwise statistical threshold of *p* ≤ 0.001 (uncorrected) with a minimum cluster extent of 10 voxels, and identified significant clusters at a clusterwise threshold of *p* < 0.05 (family wise error, FWE, corrected). As in earlier repetition suppression research^25–27,32^, simple repetition suppression effects were defined for each condition by prime > target contrasts (see weights in Table 2). We also specified main effects of prime > target contrasts for the Repeated Stereotypes combined, for the Repeated Groups combined and for the combined repetition of Groups and Stereotypes (to test a summative effect), while ignoring the non-repeated conditions (by setting the latter weights to 0; see Table 2). Finally, we also specified interactions based on the same main effects of Repeated Stereotypes and/or Groups, but now also controlling for non-repetitions in the remainder of the conditions (by setting the latter weights to 1 and adjusting the other negative weights so that the sum of weights totaled 0; see Table 2).

**Table 2:** Repetition Suppression (Prime > Target contrast) effects from the whole brain analysis with coil as between-participants factor.

Anatomical label		x	y	z	Voxels	Max t
	**Simple Prime** > **Target contrasts**					
	Repeated Group/Repeated Stereotype					
	[1 -1 0 0 0 0 0 0]					
	(no significant clusters)					
	Repeated Group/Different Stereotype					
	[0 0 1 -1 0 0 0 0]					
mPFC		2	36	14	244	3.95**
	R Superior Medial Gyrus (mPFC)	2	46	0		3.76**
	L Mid Orbital Gyrus	0	56	−2		3.46**
	Different Group/Repeated Stereotype					
	[0 0 0 0 1 -1 0 0]					
mPFC		4	42	2	466	4.67***
	mPFC	−2	34	4		3.99***
	mPFC	−2	48	−4		3.84***
	Different Group/Different Stereotype					
	[0 0 0 0 0 0 1 -1]					
	(no significant clusters)					
	**Main Suppression Effects**					
	Suppression of Group Repetition					
	[1 -1 1 -1 0 0 0 0]					
R PCC		2	−40	50	267	4.25**
	R MCC	8	−34	42		3.97**
L Mid Orbital Gyrus		0	46	−6	423	4.21***
	R Mid Orbital Gyrus	12	40	−8		3.40***
	Suppression of Stereotype Repetition					
	[1 -1 0 0 1 -1 0 0]					
R Mid Orbital Gyrus	12	40	−4	559	4.37***	
	L Mid Orbital Gyrus	−2	46	−6		4.29***
	mPFC	4	42	2		4.08***
R Supra-marginal Gyrus		60	−32	34	188	4.35*
	R Supra-marginal Gyrus	60	−36	42		4.03*
	R Inferior Parietal Lobule	56	−46	38		3.84*
	Suppression of Combined Repetition of Groups and Stereotypes					
	[1 -3 1 1 0 0 0 0]					
	(no significant clusters)					
	**Interactions**					
	Suppression for Group Repetition Only					
	[1 -3 1 -3 1 1 1 1]					
	(no significant clusters)					
	Suppression for Stereotype Repetition Only					
	[1 -3 1 1 1 -3 1 1]					
R Inferior Parietal Lobule		52	−54	48	244	4.57**
L Mid Orbital Gyrus		−2	44	−6	412	4.08***
	mPFC	4	42	2		4.07***
	mPFC	2	40	12		3.92***
	Suppression of Combined Repetition of Groups and Stereotypes Only					
	[1 -3 1 -1 1 -1 1 1]					
	(no significant clusters)					

Notes: Coordinates refer to the MNI (Montreal Neurological Institute) stereotaxic space. Whole-brain analysis thresholded at p < 0.001, uncorrected with ≥10 voxels. Reported are clusters with p < 0.05, FWE cluster-corrected. The contrast weights between parentheses refer to a pair of Prime and Target trials in the first four conditions respectively: (1) Repeated Group/Repeated Stereotype, (2) Repeated Group/Different Stereotype, (3) Different Group/Repeated Stereotype, (4) Different Group/Different Stereotype. In practice each number should appear twice in the contrast, once for the first coil and once for the second. For the sake of clarity this is not shown above.

*p < 0.05, **p < 0.01, ***p < 0.001 (FWE peak corrected).

To verify whether the brain areas identified in the interaction analysis showed the predicted repetition suppression pattern, we computed the percent signal change. Because a percent signal change analysis on the full sample cannot take a between-participant factor like coil into account, this analysis was based on ROIs derived from a second level analysis without coil as a factor (see Table 3), but to be strict, we selected only ROIs that were significant also in the second level analysis with coil as a factor. Note that this additional post-hoc test is not double dipping, but rather intends to eliminate potential false positives arising from the fMRI contrast analysis, which could result from changes in the prime instead of the target sentences. For instance, changes in activation during the prime presentation, rather than the target, could also yield significant contrasts. We identified regions of interest (ROIs) as a sphere of 3 mm around the peak coordinates of the interaction contrasts. We then extracted the percent signal change in these ROIs for each participant using the MarsBar toolbox (http://marsbar.sourceforge.net). We calculated repetition indexes for each condition, which were defined as the percentage signal change of target minus prime sentences for each condition. These data were further analyzed using t-tests with a threshold of *p* < 0.05, one-sided and uncorrected, given that these had already been limited by the whole-brain analysis.

**Table 3:** Repetition Suppression (Prime > Target contrast) effects from the whole brain analysis, without coil as between-participants factor.

Anatomical label		x	y	z	Voxels	Max t
	**Simple Prime** > **Target contrasts**					
	Repeated Group/Repeated Stereotype					
	[1 −1 0 0 0 0 0 0]					
	(no significant clusters)					
	Repeated Group/Different Stereotype					
	[0 0 1 −1 0 0 0 0]					
mPFC		2	44	−6	684	4.54***
	L Anterior Cingulate Cortex (mPFC)	2	36	14		4.16***
	L Middle Orbital Gyrus (mPFC)	−10	54	−2		3.98***
R Superior Frontal Gyrus (mPFC)		18	38	28	317	4.11**
	R Superior Frontal Gyrus	22	44	22		4.09**
	R Superior Frontal Gyrus	22	58	24		4.05**
L PCC^G^		−2	−36	44	233	4.02**
	L PCC	10	−36	42		3.68**
	Different Group/Repeated Stereotype					
	[0 0 0 0 1 −1 0 0]					
mPFC^S^		4	42	2	654	4.93***
	mPFC	0	34	4		4.32***
	mPFC	−2	48	−4		4.27***
	Different Group/Different Stereotype					
	[0 0 0 0 0 0 1 −1]					
mPFC		12	38	−12	711	5.26***
	R Middle Orbital Gyrus (mPFC)	20	38	−4		4.48***
	R Superior Medial Gyrus (mPFC)	2	54	8		4.12***
R Superior Frontal Gyrus (mPFC)		16	52	20	594	4.80***
	R Middle Frontal Gyrus	22	48	26		4.64***
	R Superior Medial Gyrus (mPFC)	6	62	28		4.51***
	**Main Suppression Effects**					
	Suppression of Group Repetition					
	[1 −1 1 −1 0 0 0 0]					
mPFC		0	44	−8	827	4.69***
	R Middle Orbital Gyrus (mPFC)	10	40	−10		4.52***
	L ACC (mPFC)	2	36	14		4.44***
R Middle Frontal Gyrus (mPFC)		24	58	24	265	4.61**
	R Superior Frontal Gyrus (mPFC)	16	62	24		4.43**
	R Superior Medial Gyrus (mPFC)	4	62	28		3.94**
L PCC		−4	−38	44	401	4.63***
	R MCC	10	−36	42		4.43***
	Suppression of Stereotype Repetition					
	[1 −1 0 0 1 −1 0 0]					
R Superior Frontal Gyrus (mPFC)		16	62	24	212	4.79*
	R Middle Frontal Gyrus (mPFC)	26	56	20		4.13*
	R Superior Frontal Gyrus (mPFC)	26	64	12		4.07*
mPFC		8	36	−8	771	4.79***
	L Middle Orbital Gyrus (mPFC)	−2	46	−8		4.75***
	R Middle Orbital Gyrus (mPFC)	6	52	−10		4.54***
	Suppression of Combined Repetition of Groups and Stereotypes					
	[1 −3 1 1 0 0 0 0]					
mPFC		10	40	−10	509	4.53***
	L Middle Orbital Gyrus (mPFC)	0	46	−8		4.18***
	L Middle Orbital Gyrus (mPFC)	−2	54	−6		3.93***
L PCC		−2	−38	44	266	4.21**
	R PCC	6	−34	42		4.12**
	**Interactions**					
	Suppression for Group Repetition Only					
	[1 −3 1 −3 1 1 1 1]					
	(no significant clusters)					
	Suppression for Stereotype Repetition Only					
	[1 −3 1 1 1 −3 1 1]					
mPFC		8	38	−12	871	4.64***
	L Middle Orbital Gyrus (mPFC)	−2	46	−8		4.61***
	mPFC ^S^	4	42	2		4.37***
R Superior Frontal Gyrus (mPFC)		14	62	24	172	4.57*
	R Middle Frontal Gyrus	24	56	22		4.38*
	R Superior Frontal Gyrus	26	64	14		3.64*
R Superior Frontal Gyrus		24	24	58	231	4.44**
	R Superior Frontal Gyrus	28	28	52		4.37**
	R Superior Frontal Gyrus	22	24	44		3.83**
	Suppression of Combined Repetition of Groups and Stereotypes Only					
	[1 −3 1 −1 1 −1 1 1]					
mPFC		10	38	−10	600	4.68***
	L Middle Orbital Gyrus (mPFC)	−2	44	−8		4.28***
	L Middle Orbital Gyrus (mPFC)	−8	52	−4		4.13***
R Middle Orbital Gyrus		24	58	24	163	4.65*
	R Superior Frontal Gyrus (mPFC)	14	62	24		4.63*
	R Superior Medial Gyrus (mPFC)	4	62	28		3.89*

Notes: Coordinates refer to the MNI (Montreal Neurological Institute) stereotaxic space. Whole-brain analysis thresholded at p < 0.001, uncorrected with ≥10 voxels. Reported are clusters with p < 0.05, FWE cluster-corrected. The contrast weights between parentheses refer to a pair of Prime and Target trials in the first four conditions respectively: (1) Repeated Group/Repeated Stereotype, (2) Repeated Group/Different Stereotype, (3) Different Group/Repeated Stereotype, (4) Different Group/Different Stereotype. Superscripts refer to the percent signal change analysis indicating suppression for Stereotypes (S) and Groups (G). mPFC = medial prefrontal cortex, L = left, R = right.

*p < 0.05, **p < 0.01, ***p < 0.001 (FWE peak corrected).

## Results

### Behavioral Results

On average a rating of 2.90 (SD = 0.78), out of a maximum of 4, was given to the applicability of the stereotypes to the groups, indicating that most sentences were considered typical. Moreover, the behavior was given the minimum rating in only 1% of the sentences. Ratings were significantly affected by both the RT of the participant, χ²(1, *N* = 39) = 88.13, *p* < 0.001, and the condition, χ²(4, *N* = 39) = 209.81, *p* < 0.001. Specifically, ratings of typicality were highest in the Repeated Group/Repeated Stereotype condition, somewhat lower in conditions in which either the group or the stereotype was repeated, but not both. It was lowest in the condition where both stereotypes and groups were different and in the singleton condition. However, note that even the lowest of these mean ratings per condition was still equal to 2.77 (SD = 0.80).

RTs averaged at 2412 ms (SD = 1561). They were significantly affected by both the stereotype-rating, χ²(1, *N* = 39) = 88.55, *p* < 0.001, and the condition, χ²(4, *N* = 39) = 26.88, *p* < 0.001. RTs tended to be very fast for a maximum rating, and very slow for minimal ratings. Moderate ratings were associated with RTs around the mean. With respect to the conditions, a typical priming effect was observed, with RTs being shortest when both groups and stereotypes are repeated and somewhat longer when only either is repeated. They were longest when both groups and stereotypes were different and in the singleton condition.

### fMRI Results

For the analysis of the fMRI data, we used a similar strategy for the detection of repetition suppression effects as in earlier research^25–27,32^. First, we conducted a whole-brain random effects analysis contrasting prime > target trials in all conditions, and then whole-brain main and interaction analyses to identify repetition effects. These analyses were followed by a signal change analysis to verify the predicted repetition suppression patterns.

### Whole-brain analysis

Using the whole-brain random effects analysis of the simple prime > target contrasts, we found significant repetition suppression effects in the mPFC in all conditions as predicted (Table 2). The main effects of the prime > target contrast for stereotypes and groups, as well as the interaction effects which additionally controlled for potential changes in the remaining non-repetition conditions by giving these conditions fixed weights, confirmed the significant repetition suppression effect in the mPFC, as well as in other areas including the frontal gyrus, mid orbital gyrus and PCC.

### Region of interest analysis

We computed the percent signal change in ROIs centered on all peak values found in the whole-brain analysis. In doing so, we can verify whether repetition suppression in these areas follows the expected suppression pattern in the target sentences because significant contrasts may arise for other reasons, such as differences in prime activation per condition. A suppression index was calculated by subtracting the percent signal change in the prime sentence from the target sentence for every condition and every ROI.

**Figure 1:**
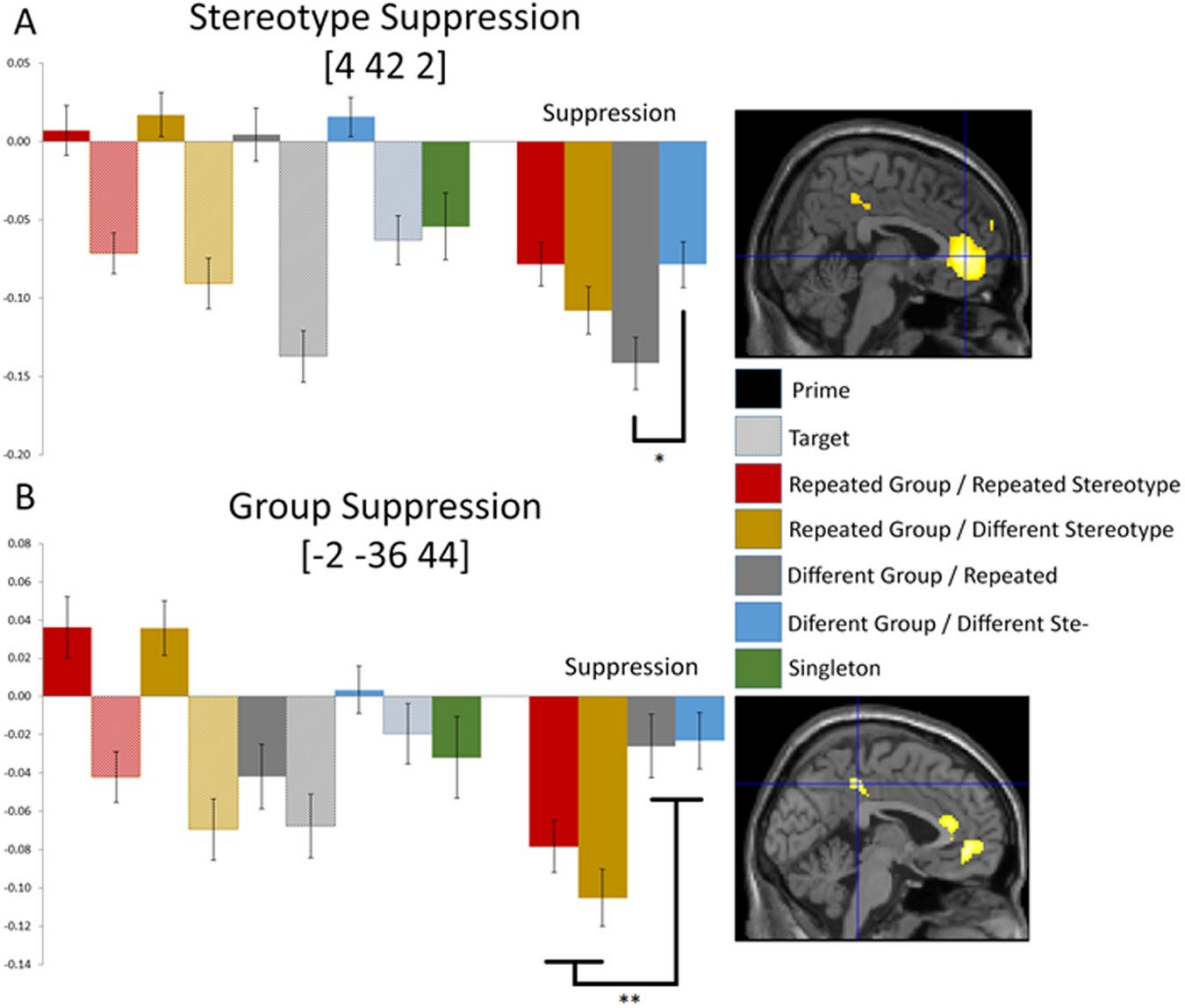
Percent signal change revealing stereotype and group suppression. The left side of each panel shows the prime and target pairs. The right side shows the suppression index. The image on the far right shows the area revealed by the whole-brain analysis, thresholded at p < 0.001 uncorrected, from which the signal change was extracted and indicated with their MNI coordinates. (**A**) Stereotype repetition suppression for different groups (suppression is significantly stronger when stereotypes are repeated (grey) than when stereotypes are not repeated; blue; *p < 0.05). (**B**) Group repetition suppression (suppression is significantly stronger for the first two conditions (red and yellow) in which groups are repeated than in the third and fourth conditions (grey and blue) when groups are not repeated; **p < 0.01).

With respect to stereotypes, the predicted pattern of repetition suppression was found in the mPFC (with MNI coordinates 4 42 2; Fig. 1A). Using a repeated analysis of variance (ANOVA) on the suppression indices of this ROI with within-participant factors Group and Stereotype, we found no significant main difference between Repeated versus Different Stereotypes, *F*(1, 38) = 0.855, *p* = 0.361. However, this could stem from the lack of a stereotype effect in the presence of repeated groups, as a paired t-test shows a significant difference between the Different Group/Repeated Stereotype and Different Group/Different Stereotype conditions, t(37) = −1.93, p = 0.03.

With respect to groups, a pattern indicating repetition suppression was found in the PCC (with MNI coordinates -2 -36 44; Fig. 1B). The repeated measures ANOVA on this ROI showed a significant main effect between Repeated versus Different Groups, F(1, 38) = 16.2, p < 0.001. Furthermore, t-tests showed that this group repetition effect was significant when stereotypes were repeated, t(37) = −2.04, p = 0.024, and when they were not, t(37) = −2.99, p = 0.002. No other repetition patterns were found in other clusters identified by the whole-brain analysis.

## Discussion

This study explored whether neural representation for groups and stereotypes exist in the brain. Based on prior research on group information processing, and earlier research on person and trait representation^25–27,32,33^, we expected to find repetition suppression reflecting neural representations for groups and stereotypes in the mPFC. The results revealed fMRI suppression for stereotypes in the mPFC. However, this suppression for stereotypes was only apparent when groups were different. In addition, repetition of group information led to suppression in the PCC, an area which was at times revealed showing suppression upon repeated processing of unfamiliar persons in earlier social repetition research^25^.

The present findings suggest that stereotypes and groups are not necessarily on-line creations derived from contextually driven patterns of activity throughout the brain^34^, but often derive from crystallized and enduring knowledge structures in specific brain areas that represent this information. This finding extends research on neural representation from the level of individual persons and traits^25–27,32,33^ to the group level. An earlier group repetition suppression study by Lau and Cikara^47^ showed suppression for ingroups in the dorsolateral prefrontal cortex and middle temporal gyrus. However, as their task involved counting the number of presented in-groups rather than making group judgments, it is unclear whether the suppression stemmed from categorizing the groups, or from counting. Moreover, it is not clear whether the effect stems from group repetition or the repetition of the self, which is included in every ingroup. The current study avoided this potential confound by revealing, for the first time, suppression for repeated outgroups. The existence of neural representations of stereotypes and groups can go a long way in explaining why humans readily use this type of social information to make rapid impressions of others, possibly leading to stereotypical and discriminatory judgments and behavior.

Our results confirm the social role of the mPFC as reservoir of social knowledge about stereotypes and traits demonstrated in previous research. However, they should be interpreted with some caution since it is still unclear why suppression for stereotypes was only revealed for groups that were different. Perhaps, the presence of a common group identity might outweigh the importance of distinct stereotypes within the same group, as the communality may hamper identification of potential differences between stereotypes revealed by the behavior of different group members. In other words, the group repetition may draw attention away from the specific behaviors group members perform. It could also be the case that, if groups are identical, the observer finds reasons for the specific behaviors to fit the overarching group identity. This could not happen if groups are different; allowing differences between stereotypes to be noticed. This finding is intuitively plausible, and extends earlier research that did not simultaneously manipulate group identity and stereotypes. Past research indeed demonstrated the role of the mPFC for processing stereotypical information in isolation, such as processing stereotypes about gender^10,31^, race^30,48–50^, and other groups in our society^51^.

Perhaps more surprising is the finding that representations of groups are not stored in the mPFC but in the PCC, despite previous research showing that processing information about groups evokes activity in the mPFC^24,51,52^. The PCC shares many of the characteristics with the mPFC that make it a likely repository for high-level social information. First, one view is that both areas have been implicated in the mentalizing network, indicating their importance in processing social information and impression formation^20,53^. In addition, a recent repetition suppression study^25^ identified suppression for unfamiliar persons in the PCC, which suggests that the unfamiliarity of the groups (i.e. outgroups) render it more likely that these social categories are stored in this area rather than in the vmPFC. And, while the mPFC is considered more as a core area in the network, the PCC has also been shown to become involved in mentalizing tasks^20^. However, the PCC is typically conceived as supporting scene construction and has been revealed in repetition suppression studies of scenes^54,55^. Together, the representation in the PCC may suggest that outgroups are perhaps seen more as part of a less familiar or less close social background rather than a collective of individuals (who consistently show suppression in the mPFC). Second, a complementary view is that both areas are involved in an extended semantic network^56^, where they receive extensively processed, multimodal input that makes them important for high-level integrative processes^21^. As such, both are good candidates for abstract information representation, such as groups. This is also important given the recent finding that social groups are processed separately from the typical living and non-living categories in the semantic system^57^. In addition to these commonalities, the PCC is sensitive to information that is considered motivationally relevant for valuation^58^. This is especially the case for stimuli that are of a social rather than non-social nature^59^. The presence of group representations in the PCC could thus be a reason for the readiness with which group identity is used to judge others.

There are a number of alternative explanations for our findings that are worth considering. First, the current study did not take into account the valence of the presented behaviors, nor of the groups. The reason is that valence was often not clearly positive or negative, but also relatively neutral. Moreover, depending on the group and situation, the same behaviors could be seen as positive (a talkative child) or negative (a talkative colleague). By not controlling for valence, it is possible that repetition suppression reflects a reaction to similar valence, rather than similar groups or stereotypes. However, this is unlikely. To exclude valence as an explanation for trait repetition suppression, Ma and colleagues^32^ set up an experiment in which they manipulated the valence of traits (of persons) and features (of objects). They found suppression in response to repetition of traits, but not given trait - feature sequences of similar valence. Thus, repetition suppression was limited to personality traits, and did not extend to general valence. A second explanation could be that our stereotype repetition suppression reflects a process of abstraction rather than a neural code for specific stereotypes^23,60,61^. However, non-repetition of stereotypes was created by presenting different stereotypes in the two sentences. Under these conditions, although abstraction is required for both sentences, suppression was not found. Third, it is possible that the areas found are only involved in processing groups and stereotypes and not their representation^41,61,62^. Suppression after repetition would then only be due to the earlier activation, which does not need to involve any representation. This interpretation, however, fails to explain why such activation leads to suppression in some limited areas and not in other areas, as there is typically much more activation going on.

Future studies that aim to localize a group representation should avoid individualization of the protagonists to obtain stronger effects. Moreover, they should also avoid the inclusion of the self (as a confounding factor) in the presented group^47^. Of more interest, future research should look into different subtypes of groups to see whether they are all processed in the same area. In a study by Harris and Fiske^51^ the researchers found that groups low on warmth and competence (e.g., drug addicts, homeless) activated the mPFC significantly less than other groups Perhaps, these groups differing on these dimensions might be represented differently in the brain as well.

## Conclusion

Our present findings support the idea that there are neural representations of group stereotypes in the mPFC, which are however revealed only for distinct groups and should be investigated further. Moreover, we demonstrated that groups are represented in the PCC. Both brain areas are important for social mentalizing and have previously been linked to representation of other social information such as persons and their characteristics. However, more research is required to corroborate our findings and to extend it to specific (sub)types of groups.
