# Author Correction: Neural representations of Groups and Stereotypes using fMRI repetition suppression

**DOI:** 10.1038/s41598-020-60916-4

**Published:** 2020-02-26

**Authors:** Jeroen Delplanque, Elien Heleven, Frank Van Overwalle

**Affiliations:** Vrije Universiteit Brussel, Brussel, Belgium

Correction to: *Scientific Reports* 10.1038/s41598-019-39859-y, published online 28 February 2019

The Article contains errors in Table 2, where there is a missing column named “Effect type”.

As a result, the subtitles “Simple Prime > Target contrasts”, “Main Suppression Effects” and “Interactions” originally under the column “Anatomical label” should be found under the column “Effect type”.

The correct Table 2 appears below as Table 1

**Table 1:** .

Effect type	Anatomical label	x	y	z	Voxels	Max t
**Simple Prime > Target contrasts**						
	**Repeated Group/Repeated Stereotype**					
	[1 -1 0 0 0 0 0 0]					
	(no significant clusters)					
	**Repeated Group/Different Stereotype**					
	[0 0 1 -1 0 0 0 0]					
	mPFC	2	36	14	244	3.95**
	R Superior Medial Gyrus (mPFC)	2	46	0		3.76**
	L Mid Orbital Gyrus	0	56	−2		3.46**
	**Different Group/Repeated Stereotype**					
	[0 0 0 0 1 -1 0 0]					
	mPFC	4	42	2	466	4.67***
	mPFC	−2	34	4		3.99***
	mPFC	−2	48	−4		3.84***
	**Different Group/Different Stereotype**					
	[0 0 0 0 0 0 1 -1]					
	(no significant clusters)					
**Main Suppression Effects**						
	**Suppression of Group Repetition**					
	[1 -1 1 -1 0 0 0 0]					
	R PCC	2	−40	50	267	4.25**
	R MCC	8	−34	42		3.97**
	L Mid Orbital Gyrus	0	46	−6	423	4.21***
	R Mid Orbital Gyrus	12	40	−8		3.40***
	**Suppression of Stereotype Repetition**					
	[1 -1 0 0 1 -1 0 0]					
	R Mid Orbital Gyrus	12	40	−4	559	4.37***
	L Mid Orbital Gyrus	−2	46	−6		4.29***
	mPFC	4	42	2		4.08***
	R Supra-marginal Gyrus	60	−32	34	188	4.35*
	R Supra-marginal Gyrus	60	−36	42		4.03*
	R Inferior Parietal Lobule	56	−46	38		3.84*
	**Suppression of Combined Repetition of Groups and Stereotypes**					
	[1 -3 1 1 0 0 0 0]					
	(no significant clusters)					
**Interactions**						
	**Suppression for Group Repetition Only**					
	[1 -3 1 -3 1 1 1 1]					
	(no significant clusters)					
	**Suppression for Stereotype Repetition Only**					
	[1 -3 1 1 1 -3 1 1]					
	R Inferior Parietal Lobule	52	−54	48	244	4.57**
	L Mid Orbital Gyrus	−2	44	−6	412	4.08***
	mPFC	4	42	2		4.07***
	mPFC	2	40	12		3.92***
	**Suppression of Combined Repetition of Groups and Stereotypes Only**					
	[1 -3 1 -1 1 -1 1 1]					
	(no significant clusters)					

